# Prolonged Complete Response in a Patient with Metastatic Pancreatic Adenocarcinoma after FOLFIRINOX Chemotherapy and Maintenance with FOLFIRI

**DOI:** 10.1155/2015/659624

**Published:** 2015-05-21

**Authors:** Christos Nikolaou, Alexios Matikas, Maria Papavasilopoulou, Dimitris Mavroudis, Lampros Vamvakas

**Affiliations:** ^1^Department of Medical Oncology, University Hospital of Heraklion, Voutes, P.O. Box 1352, Heraklion, 71110 Crete, Greece; ^2^Olympion General Clinic, Volou & Meilichou, 26443 Patra, Greece

## Abstract

Metastatic pancreatic adenocarcinoma confers a poor prognosis. Even with recent advances in the treatment of this disease with the introduction of two modestly effective chemotherapy regimens, complete responses are still very rare. Moreover, there are no published data on how to further manage the patients who achieve a sustained remission following treatment. Herein, we report the case of a patient with metastatic pancreatic adenocarcinoma who achieved a complete response lasting for more than three years after receiving induction chemotherapy with FOLFIRINOX followed by maintenance with FOLFIRI.

## 1. Introduction

Primary adenocarcinoma of the pancreas is the eighth most common cancer and fourth leading cause of cancer-related death in the United States; the vast majority of newly diagnosed patients will eventually die of the disease [1]. Even when amenable to “curative” surgery, most patients eventually relapse. Despite recent advances, metastatic pancreatic cancer remains a devastating disease and carries a grim prognosis, with less than 1% of patients surviving for more than 5 years [1].

Until 2011, monotherapy with gemcitabine was the standard of care leading to an overall survival (OS) of approximately 6 months and a one-year survival rate of less than 20%; objective radiographic responses were rare [2]. Gemcitabine has been combined with a variety of cytotoxic and targeted agents, such as oxaliplatin [3], capecitabine [4, 5], 5-fluorouracil [6], irinotecan [7], and erlotinib [8]. However, the improvement in OS has been either statistically or clinically insignificant. In 2011, the results of the ACCORD 11 trial were published, which showed that the combination of 5-fluorouracil, irinotecan, and oxaliplatin (FOLFIRINOX) produced superior response rate, progression-free survival (PFS), and OS compared to single agent gemcitabine [9]. More recently, the combination of gemcitabine plus nanoparticle albumin bound paclitaxel (nab-paclitaxel) was also shown to be superior in terms of OS compared to gemcitabine [10]. Despite the aforementioned advances, almost all patients with metastatic pancreatic cancer eventually die of progressive disease, underscoring the clearly unmet need of curative therapy and the lack of data on how to best sequence existing active regimens when treating these patients.

In this case report, we present the case of a 51-year-old man with lung metastases from pancreatic adenocarcinoma who achieved a complete response after initial chemotherapy with FOLFIRINOX followed by maintenance chemotherapy with irinotecan, leucovorin, and 5-fluorouracil (FOLFIRI) and who has not yet recurred after 3 years from the initial diagnosis of metastatic disease. We propose this approach for highly selected patients with sustained responses to chemotherapy.

## 2. Case Presentation

A 51-year-old male patient with a medical history of type II diabetes and a smoking history of over 30 pack-years presented with a 2-month history of worsening epigastric pain, jaundice, and weight loss of approximately 10 kg. Physical examination was otherwise unremarkable. An abdominal computed tomography (CT) scan revealed a mass at the head of the pancreas with a maximal diameter of 2.4 cm which caused dilatation of the pancreatic and biliary tract. Further testing revealed markedly elevated levels of CA 19-9 tumor marker exceeding 12000 U/mL (normal range: 0–37 U/mL). Subsequent staging with chest CT and abdominal magnetic resonance imaging (MRI) showed absence of metastases and the patient underwent a Whipple procedure. The pathology exam was diagnostic of a primary pancreatic adenocarcinoma with a maximal diameter of 3.2 cm, with perivascular and perineural invasion. Four out of seven excised peripancreatic lymph nodes were positive for metastatic disease and all surgical margins were negative. According to the American Joint Committee on Cancer (AJCC) staging system, pathologic stage was pT2N1M0, stage II.

The patient's recovery was uneventful. Postsurgery CT scans showed no evidence of metastatic or residual disease and CA 19-9 tumor marker levels were 1203 U/mL. Adjuvant chemotherapy with gemcitabine 1000 mg/m^2^ on days 1, 8, and 15 on 28-day cycles was started 2.5 months after surgery, with a plan of administering 6 cycles. After completing 2 cycles, a gradual increase of CA 19-9 levels was noted and new imaging was performed. New multiple bilateral pulmonary nodules as well as para-aortic lymph nodes and one new liver lesion were shown, findings consistent with metastatic disease. On physical examination the patient remained asymptomatic, with an Eastern Cooperative Oncology Group (ECOG) performance status of 0. A palpable left supraclavicular lymph node approximately 1.5 cm in diameter was noted. CA 19-9 levels were increased to over 12000 U/mL.

**Figure 1:**
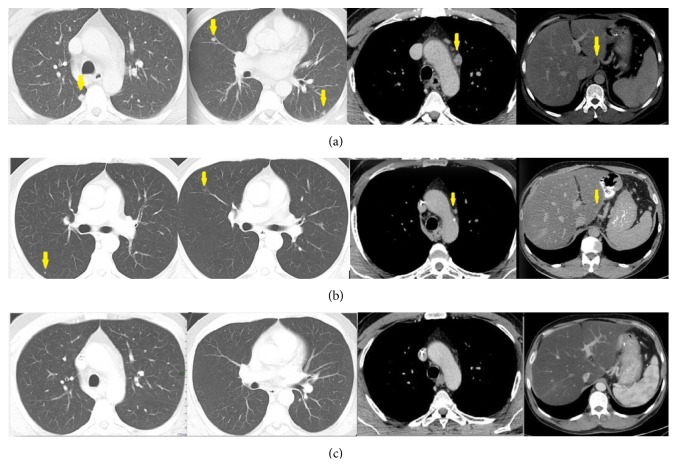
CT scan, axial images, at the same level, at the beginning of the therapy (a), after three cycles of chemotherapy (b) showing partial response (PR), and after the seventh cycle (c) in competence with complete response (CR). Yellow arrows indicate the pulmonary nodules (first two images) that are easily recognized at the first scan, are faintly differentiable at the following, and have disappeared at the final. In the remaining images, arrows indicate the pathologic mediastinal lymph node (third image) and the liver lesion.

First-line chemotherapy with the combination of oxaliplatin 85 mg/m^2^, irinotecan 180 mg/m^2^, leucovorin 400 mg/m^2^, and fluorouracil 400 mg/m^2^ given as a bolus followed by 2400 mg/m^2^ given as a 46-hour continuous infusion, every 2 weeks (FOLFIRINOX), was initiated. After 4 administrations of chemotherapy a gradual decrease of CA 19-9 levels to 550 U/mL was evident, while the left supraclavicular lymph node was no longer palpable. Imaging revealed a partial response of the pulmonary nodes and a complete response of the para-aortic lymph nodes and liver lesion (Figure 1). After 4 more administrations (8 cycles in total) the patient attained a complete response which he maintained after seven months of FOLFIRINOX chemotherapy despite dose reductions due to hematologic toxicity. Because of worsening neurotoxicity caused by oxaliplatin which peaked at grade 3 and taking into account the absence of published data regarding the optimal management of this patient, maintenance chemotherapy with FOLFIRI was commenced. Due to sustained complete remission on reimaging studies, the patient completed a total of two years of chemotherapy and discontinued treatment due to steatohepatitis with grade 1 transaminitis probably caused by irinotecan. Eight months after treatment discontinuation he remained alive and in complete radiographic and biochemical remission.

## 3. Discussion

In spite of the use of more active combination regimens, complete responses are a rare occurrence in metastatic pancreatic cancer; in the ACCORD 11 trial, there was only one complete response among the 171 patients who received FOLFIRINOX (0.6%) [9]. Published case series propose total gross resection for locally advanced pancreatic cancer following dramatic responses from first-line chemotherapy [11, 12]. Complete radiological or pathologic complete responses after neoadjuvant FOLFIRINOX are rare but well documented in this setting [13–15]; these patients may have a better prognosis compared to those who experience lesser responses [16]. However, there is no published literature on how to best treat the rare patient with distant metastases who achieves a sustained partial or complete response, due to the rarity of such cases [17]. Current guidelines support the continuation of intensive chemotherapy until disease progression or unacceptable toxicities, as was the case with our patient [18]. Also, the role of Positron Emission Tomography (PET and PET/CT) scan in the initial staging of pancreatic cancer is controversial and there are no data to support its use in the metastatic setting [19].

Taking into account the absence of measurable disease after achieving a complete response and grade 3 neurotoxicity which affected our patient's activities of daily living and his unwillingness to stop treatment altogether, we proposed omitting oxaliplatin and continue with FOLFIRI. To our knowledge, this is the first published report of maintenance FOLFIRI after initial response to FOLFIRINOX in a patient with pancreatic cancer. How much benefit the patient actually derived from FOLFIRI cannot be determined, especially in light of the modest activity of irinotecan in metastatic pancreatic cancer [20].

In recent years, the traditional approach of distinct chemotherapy lines when treating metastatic solid tumors is being challenged in favor of a continuum of care paradigm where periods of intensive chemotherapy are followed by less toxic maintenance strategies. Whether such an approach can be successfully implemented in the treatment of metastatic pancreatic cancer remains to be seen in future trials. In addition, the optimal sequence of active regimens and whether surgical excision of metastatic sites in patients who exhibit significant response to systemic treatment has a role in the treatment strategy are questions that also remain unanswered, although the feasibility of the latter strategy has been demonstrated in case reports [21].

In conclusion, we present the case of a patient with prolonged complete response after initial chemotherapy with FOLFIRINOX and maintenance with FOLFIRI. Until data from prospective randomized trials are available and taking into account the absence of any relevant published literature, case reports and retrospective case series may offer a guide on how to treat the selected population of long-term (i.e., more than two years) survivors with metastatic pancreatic cancer.
